# Suicide Seasonality: Complex Demodulation as a Novel Approach in Epidemiologic Analysis

**DOI:** 10.1371/journal.pone.0017413

**Published:** 2011-02-24

**Authors:** Ingo W. Nader, Jakob Pietschnig, Thomas Niederkrotenthaler, Nestor D. Kapusta, Gernot Sonneck, Martin Voracek

**Affiliations:** 1 Department of Basic Psychological Research, School of Psychology, University of Vienna, Vienna, Austria; 2 Department of General Practice and Family Medicine, Center for Public Health, Medical University of Vienna, Vienna, Austria; 3 Department of Medical Psychology, Center for Public Health, Medical University of Vienna, Vienna, Austria; 4 Department of Psychoanalysis and Psychotherapy, Medical University of Vienna, Vienna, Austria; 5 Ludwig Boltzmann Institute for Social Psychiatry, Vienna, Austria; University of Michigan, Canada

## Abstract

**Background:**

Seasonality of suicides is well-known and nearly ubiquitous, but recent evidence showed inconsistent patterns of decreasing or increasing seasonality in different countries. Furthermore, strength of seasonality was hypothesized to be associated with suicide prevalence. This study aimed at pointing out methodological difficulties in examining changes in suicide seasonality.

**Methododology/Principal Findings:**

The present study examines the hypothesis of decreasing seasonality with a superior method that allows continuous modeling of seasonality. Suicides in Austria (1970–2008, *N* = 67,741) were analyzed with complex demodulation, a local (point-in-time specific) version of harmonic analysis. This avoids the need to arbitrarily split the time series, as is common practice in the field of suicide seasonality research, and facilitates incorporating the association with suicide prevalence. Regression models were used to assess time trends and association of amplitude and absolute suicide numbers. Results showed that strength of seasonality was associated with absolute suicide numbers, and that strength of seasonality was stable during the study period when this association was taken into account.

**Conclusion/Significance:**

Continuous modeling of suicide seasonality with complex demodulation avoids spurious findings that can result when time series are segmented and analyzed piecewise or when the association with suicide prevalence is disregarded.

## Introduction

Seasonality of suicides is a long-known and well-established phenomenon, with investigations published as early as 1825 [1]. Research showed that the pattern of seasonality is sex-specific: For men, studies showed an annual cycle with a single peak in spring, while for women, most (but not all) studies also revealed an additional biannual cycle with a smaller second peak in autumn [2], [3]. Furthermore, seasonality has been found to be confined to violent suicide methods (i.e., all methods except poisoning [4]), specifically to hanging and drowning [5], and sometimes to jumping from high places [6].

Already in 1981, seasonal fluctuations in suicidality were suspected to be diminishing in Britain [7]. More recently, seasonality of suicide was reported to be decreasing in Finland [8], Denmark [9], the Swedish island of Gotland [10], England and Wales [11], and Switzerland [5]. Other studies found no decrease in seasonality in Italy [12], and even an increase in Australia [13] and the USA [14]. It was hypothesized that absolute suicide numbers are related to the strength of seasonality in suicides [15], which, from a mathematical point of view, is a logically consistent assumption: When the absolute suicide frequency is higher, seasonal variation increases. This hypothesis has been partly confirmed by two previous studies [16], [17].

Another, less disputed (but also not as intensively investigated) change in suicide seasonality relates to the location of the peak in the annual cycle. Although it is generally accepted that suicide rates are highest in spring or early summer, a Danish study reported that the peak occurs earlier now than a hundred years ago [18].

Research into suicide seasonality has employed a variety of statistical methods [19], ranging from simple Chi-squared tests to more complex methods such as harmonic analysis or spectral analysis. These latter two procedures are standard methods for the analysis of seasonal time-series data and aim to identify the contributions of different frequencies (cycles per year) in the periodic patterns in the time-series data. They are well suited for this purpose, but are unable to assess changes in cyclic patterns over time. Therefore, most studies have investigated changes in seasonality by arbitrarily splitting time series into two or more parts and comparing the respective findings. This entails serious drawbacks. First and foremost, splitting the time series is not based on any theoretical assumption. Moreover, doing so might obscure important aspects or produce spurious findings.

A much more appropriate way is to employ statistical techniques that allow continuous estimation of the relevant variable (e.g., amplitude), like complex demodulation [20]. This method estimates amplitude and phase (location of peak) of a time series for each time point of the study period. To the authors' knowledge, complex demodulation has not yet been applied in the field of suicide seasonality research.

We aimed at demonstrating the benefits of this technique by applying it to suicide statistics of the Austrian population. We explored potential shifts in the location of the peak and possible changes in the strength of suicide seasonality (amplitude), taking into account a possible relation of amplitude and absolute suicide numbers.

## Methods

### Study Sample

We obtained daily suicide data in the period from January 1, 1970, to December 31, 2008, from Statistics Austria. In this period, 67,741 suicides were recorded (71.4% vs. 28.6% by men vs. women). Suicide method was coded by ICD-8, ICD-9, and ICD-10, depending on the date of the recorded death.

### Data Preparation

**Figure 1:**
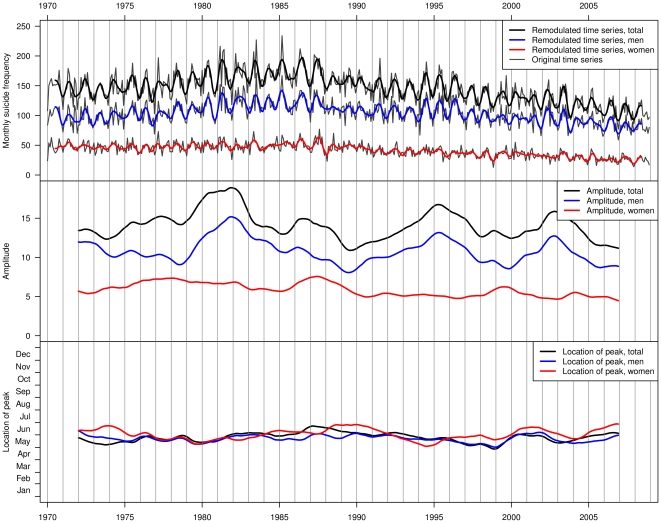
Monthly suicide numbers and complex demodulation results. Upper panel shows the remodulated and original time series of absolute suicide frequencies for the total sample, men, and women. Middle panel depicts the amplitude as a function of time; amplitude is the difference of monthly suicide numbers from the deseasonalized yearly trend at a specific point in time. Lower panel shows the location of the peak (phase) as a function of time. For clarity of presentation, amplitude and phase plots are additionally smoothed by a 37-month centered moving average.

From the daily suicide data, we calculated monthly suicide numbers. To account for the unequal length of months, these were adjusted to a standard month of 30.44 (365.25/12) days. For analysis, the time series' trend had to be removed. As the trend changed within the study period (increasing suicide rates until 1986, whilst decreasing rates thereafter; see Figure 1), we estimated the yearly trend by means of a thirteen-month centered moving average [21]. This procedure was chosen for ease of interpretation, as using an even number of months would induce a shift of the resulting average by half a month. The first month (e.g., first year's January) and the thirteenth month (e.g., second year's January) were weighted with 0.5; thus, all twelve months of the year had the same weights and the resulting average can be interpreted as the average monthly suicide frequency in a one-year period around the respective month.

### Data Analysis

To assess the relative contributions of all possible frequencies in suicide seasonality, a periodogram analysis [22] was performed. This method estimates the amount of variance explained by every possible frequency in the time series (and is the unsmoothed variant of spectral analysis). We used Fisher's *g* test [23] to check for significant seasonality, and relevant frequencies (explaining significant parts of the overall variation) were revealed by using the tables of Russell [24]. In accordance with previous studies (e.g., [6], [25]), this was performed for the whole sample, for men and women separately, and finally for different suicide methods (hanging, drowning, shooting, jumping from high places, poisoning, cutting, and other methods) for men and women, respectively.

When significant one-year cycles were found, we used complex demodulation to assess changes in amplitude and phase (location of peak). Complex demodulation may be regarded as a local version of harmonic analysis [20], whereby the frequency is fixed at a certain value (in this case, one cycle per year) and amplitude and phase are allowed to vary. This yields two new time series: one for the amplitude at each time-point of the study period, and another one for the phase. These resulting time series are per definition rough and fluctuating and thus have to be filtered; hence, smoothing is an inherent feature of this data-analytic method. This was accomplished by applying another thirteen-month centered moving average. Furthermore, the smoothed amplitude and phase time series may be used to reconstruct (i.e., remodulate) the key features of the original time series. This remodulated time series can then be compared to the original data to assess fit.

To evaluate whether amplitude and phase changed over time, we used linear regression models. As the amplitude has been found [12], [16] to be associated with absolute suicide numbers, suicide frequency was included as a predictor. Because assumptions of the linear model are generally violated in time-series data due to autocorrelated residuals, significance testing was performed using an appropriate covariance estimator [26], [27]. All analyses were performed in the statistical software package R (Version 2.10.1) [28].

## Results

### Overview

The first subsection presents an overall analysis of seasonality, without taking into account possible changes during the study period. We used periodogram analysis [22] to explore general patterns in suicide seasonality in the Austrian population.

The second subsection aims at revealing dynamic changes in suicide seasonality. This is achieved by complex demodulation [20], which estimates amplitude and location of peak continuously from the original time series of monthly suicide frequencies. To assess time trends in these variables, we applied regression models.

### Periodogram Analysis of Seasonality

In the total sample, an annual cycle explained 29.8% of variance. We found seasonal patterns to be stronger for men: In men, 26.1% of variance was explained by an annual cycle, whereas in women, seasonality was less pronounced. The annual cycle explained 14.3% of variance, and, consistent with previous research [7], an additional biannual cycle was found, explaining further 4.6%. Moreover, we found a periodic component with a frequency of 6 cycles per year in the total sample and in women, as well as some significant non-seasonal frequencies at non-integer frequencies, but each of them accounted for less than 2.7% of variance (Table 1).

**Table 1:** Suicide seasonality for the total sample, men and women (all methods).

	Frequency	Variance explained	*p* value
Total sample			<0.001
	1.00	29.8%	
	6.00	2.7%	
Men			<0.001
	1.00	26.1%	
	5.23	2.6%	
Women			<0.001
	1.00	14.3%	
	2.00	4.6%	
	1.74	2.6%	
	6.00	2.2%	
	1.59	2.1%	

Results of periodogram analysis (significant frequencies and the proportion of variance explained by that frequency) and Fisher's *g* test of significant seasonality against the null hypothesis of no seasonality. Frequency is the number of cycles per year, *p* values are one-sided.

For men, only the methods hanging and drowning showed significant annual seasonality. Annual cycles explained 27.2% of variance for the method of hanging. No significant biannual cycle was found, but there were a number of non-seasonal frequencies (Table 2). Drowning also revealed marked annual cycles (17.8%), and no other frequencies contributed significantly. For shooting, jumping from high places, poisoning, cutting, and other suicide methods, no significant annual cycles were found for men.

**Table 2:** Suicide seasonality for specific suicide methods for men and for women.

	Men			Women		
	Frequency	Variance explained	*p* value	Frequency	Variance explained	*p* value
Hanging			<0.001			<0.001
	1.00	27.2%		1.00	5.9%	
	1.36	3.6%		2.00	4.4%	
	2.85	2.4%		1.58	4.2%	
	5.23	2.2%		1.31	2.4%	
Drowning			<0.001			<0.001
	1.00	17.8%		1.00	9.0%	
				6.00	3.4%	
Shooting			0.071			0.037
				1.41	3.7%	
Jumping			0.004			<0.001
	1.67	4.7%		1.00	6.8%	
	1.23	4.1%		3.97	2.9%	
	1.00	3.4%		1.08	2.6%	
	1.28	2.6%		4.18	2.4%	
				1.21	2.1%	
Poisoning			0.005			0.730
	1.38	4.4%				
	3.00	2.8%				
Cutting			0.708			0.357
Other			0.260			0.556

See Table 1.

For women, annual patterns of seasonality were found for the methods of hanging, drowning, and (in contrast to men) jumping from high places. Seasonality was strongest for drowning, where the annual cycle explained 9.0% of the variance. For hanging and jumping from high places, annual patterns explained 5.9% and 6.8%, respectively.

Women who died by hanging were the only subgroup that showed a significant biannual cycle (4.4%). We found no significant annual or biannual seasonality for shooting, poisoning, cutting, or other methods (Table 2).

As expected, hanging was found to be the most common suicide method in our sample, and suicide incidence was much higher for men (Table 3). Therefore, seasonal patterns of the total sample were most likely to be driven by the method of hanging, especially in men.

**Table 3:** Frequencies (absolute and relative) of specific suicide methods in Austria.

Method	Men		Women		Total	
	Frequency	Percent	Frequency	Percent	Frequency	Percent
Hanging	24356	50.4%	7056	36.4%	31412	46.4%
Drowning	1341	2.8%	2159	11.1%	3500	5.2%
Shooting	9609	19.9%	507	2.6%	10116	14.9%
Jumping	3072	6.4%	2853	14.7%	5925	8.7%
Poisioning	5980	12.4%	4951	25.6%	10931	16.1%
Cutting	1060	2.2%	369	1.9%	1429	2.1%
Other	2954	6.1%	1474	7.6%	4428	6.5%

### Complex Demodulation of Seasonality

Although the cyclic pattern is only explained by a single frequency (i.e., an annual cycle), the remodulated time series captured the main features of the original time series adequately (Figure 1, upper panel). Percentage of explained variance ranged from 48.4% for the total sample to 26.3% for hanging in women (Table 4, first column).

**Table 4:** Relation of amplitude to absolute suicide numbers and time trends in amplitude of suicide seasonality.

	Variance explained	*r*_amp,abs_	Regression			
			Absolute number	*p*	Time	*p*
Total sample	48.4%	0.33	0.060	0.026	0.011	0.769
Men, all methods	47.4%	0.32	0.060	0.023	−0.026	0.381
Men, hanging	45.2%	0.53	0.214	<0.001	0.051	0.154
Men, drowning	36.3%	0.52	0.326	<0.001	−0.009	0.040
Women, all methods	30.3%	0.48	0.076	0.045	−0.001	0.959
Women, hanging	26.3%	0.46	0.000	1.000	−0.043	0.025
Women, drowning	30.4%	0.54	0.225	0.001	−0.002	0.794
Women, jumping	25.4%	0.47	0.240	<0.001	0.004	0.227

Variance explained is the squared correlation of the (detrended) original time series and the remodulated time series from complex demodulation. *r*_amp,abs_ is the correlation of amplitude and absolute suicide frequency (deseasonalized yearly trend, estimated by 13-month centered moving average). Absolute number is the change in amplitude (in absolute number of suicides) when the absolute suicide frequency (deseasonalized yearly trend) increases by one suicide. Time is the linear change in amplitude per year (in absolute number of suicides) when absolute monthly suicide frequency is held constant.

Trends of amplitude and phase (location of peak) are also shown in Figure 1 (middle and lower panel). Amplitude is the deviation of monthly suicide numbers from the deseasonalized yearly trend at a specific time. The difference of lowest and highest suicide frequencies in the course of a year therefore equals twice the amplitude.

The amplitude of the total sample was markedly unstable during the study period and mainly driven by men. The latter is not surprising, since suicide incidence was much higher in men as compared to women. The amplitude was lower in women, showing less variation over time. Mere visual inspection suggested a relation between absolute suicide numbers and amplitude: When comparing the yearly trend of the original series with the amplitude obtained from complex demodulation (Figure 1, upper panel), it is obvious that the strength of suicide seasonality (i.e., amplitude) was higher when absolute suicide numbers were high, and that the amplitude decreased when absolute suicide numbers went down. Correlations between amplitude and deseasonalized yearly trend of absolute suicide numbers ranged from *r* = .32 to *r* = .54 in subsamples where adequate annual seasonality was identified by periodogram analysis (Table 4, second column).

For a more formal approach, we applied linear regression models, where the amplitude was predicted by two explanatory variables: absolute suicide frequency (deseasonalized yearly trend, estimated by 13-month centered moving average) and time. Results showed that absolute suicide numbers were related to the amplitude in the total sample as well as in all other subsamples with significant annual seasonality (Table 4), except in the subsample of women that used hanging as suicide method. In this subgroup, no trend was present. Consequently, significance of the relation between amplitude and absolute suicide numbers could not be assessed due to lack of variation in the explanatory variable.

Regarding time trends in the strength of suicide seasonality (that is, amplitude), no decreasing or increasing seasonality was found in the total sample and also in most subsamples. The only subgroups that showed a slight, but nominally significant, decrease of seasonality were men who died by drowning and women who used the method of hanging.

Regarding time trends in the location of the peak, similar regression models were fitted, including time as explanatory variable. We found no significant changes in the location of maximum suicide incidence in any subsample (only subsamples with significant annual seasonality were considered; all *p*s>0.08, further results omitted for brevity). Despite showing no significant time trends, location of the peak showed considerable variation during the study period, ranging from mid-March to August. Figure 1, which was smoothed for clarity, still shows variation from late April to late June. On average, the peak location was in late May.

## Discussion

The major goal of this study was to investigate the hypothesis of decreasing seasonality with new and appropriate methodology. We showed that continuous modeling of amplitude, obtained by complex demodulation, is an adequate and convenient way of investigating changes in strength of suicide seasonality. We also demonstrated that the strength of seasonality is associated with absolute suicide numbers. When this association was taken into account, no changes in strength of suicide seasonality were found.

### Methodological Aspects in Suicide Seasonality Research

In our study, we used complex demodulation to obtain a continuous estimate of amplitude during the study period. Other studies segmented their time series and compared the results for different time periods. In most (if not all) cases, this segmentation was performed arbitrarily and was not theoretically founded. Furthermore, this approach has methodological disadvantages. On the one hand, it might obscure important aspects because it is not fine-grained enough, and on the other hand it might produce spurious findings when fluctuations in amplitude are large (as was seen in our results) or when segments of the time series are too short.

Moreover, a continuous estimate of amplitude over time, as obtained by complex demodulation, facilitates taking into account possible associations of strength of seasonality (amplitude or ratio of seasonal variation) with absolute suicide numbers. This effect has been disregarded in the majority of studies examining changes in suicide seasonality, which in turn might also have led to artificial results. This does not only apply to the amplitude of seasonal variation obtained from complex demodulation, but also to another method frequently used in suicide seasonality research: In harmonic analysis, the fraction of variance attributed to seasonal patterns is known to be proportional to the overall mean in the specific period [29]. Hence, changes in seasonality are influenced by changes in absolute suicide numbers and might give rise to spurious results.

### Association of Amplitude and Absolute Suicide Numbers

The present study supported the hypothesized association of suicide seasonality strength and absolute suicide numbers in the Austrian population. This link has also been demonstrated in other studies [12], [16]. Therefore it seems reasonable that this is a generalizable finding and that this effect is also present in other countries.

As our findings indicate a moderate correlation of up to .54 between the amplitude and absolute suicide numbers, a substantial fraction of suicides do not seem to show seasonal variation. This has already been hypothesized by prior research: Possible explanations include that only suicides related to psychiatric illness are seasonal [9] and that the decrease in seasonality of suicides corresponds to an increase in antidepressant prescription numbers [10]. However, we found no decrease in seasonality despite documented strong increases of antidepressant prescriptions in Austria [30]. Seasonality of suicides has also been shown to be more pronounced in rural than in urban contexts [5], offering another potential explanation. The present study cannot confirm or disprove any of these possible explanations, but the results do strengthen the hypothesis that only a proportion of suicides show seasonal variation.

### Changes in the Strength of Suicide Seasonality

Seasonality of suicides has been shown to decrease in most [5], [8]–[11], but not all, countries [13], [14]. Furthermore, the amplitude of seasonal variation has repeatedly been shown to be associated with absolute suicide numbers [12], [16], which also held with the present data. This latter effect may well account for the inconclusive evidence from comparisons of different countries. In Finland, where a slight decrease in seasonality was found, absolute suicide numbers also decreased for violent suicides in the last years of the study period. The authors of that study already suspected these facts to be related, but did not address this matter in further analysis [8]. Similar patterns have been reported in other studies [5], [9]–[11], [31]. In Australia, on the other hand, both seasonality and absolute suicide numbers increased [13], as well as in the USA [14].

Therefore, the association of seasonal variation and absolute suicide numbers might be an adequate link to integrate seemingly heterogeneous and directionally opposite findings concerning changes in suicide seasonality across countries. We hypothesize that changes in suicide seasonality may turn out to be smaller and much more consistent across different nations than currently is widely believed, once this association is considered.

### Suicide Seasonality in Austria

Putting the results of this study into the context of other findings requires some explication, as prior related studies used a variety of distinctly different methods. Chi-squared tests do not report explained variance and for this reason are not comparable with more advanced methods. In harmonic analysis, the total variance is split into seasonal harmonics (integer frequencies), non-seasonal harmonics (non-integer frequencies), and random variation. Contributions of specific frequencies (e.g., a one-year cycle) are sometimes reported analogously to the results of periodogram analysis, but at other times as percentage within seasonal harmonics, which themselves are a percentage of total variance. In this case, the contribution of a certain frequency is obtained by multiplication of the two percentages, which can then be compared to the present study's results.

One-year cycles explained 29.8% of variance in our sample, which is comparable to the findings of other studies (e.g., 31 to 44% in the USA [14]). For men vs. women, we found the one-year cycle to explain 26.1 vs. 14.3% of variance, respectively, which is also comparable to results of other countries (e.g., 16 to 39% vs. 11 to 37% in Italy [12], and 20 to 39% vs. 3 to 18% in Slovenia [32]). We found seasonality to be confined to hanging and drowning (in men and women) and jumping from high places (in women only), which similarly is consistent with studies analyzing specific suicide methods [5], [6]. Also conforming to prior research, we detected a biannual cycle in women [7], and we found this biannual cycle only for hanging.

Our results did not support a shift in the peak of suicide incidence. It has to be noted that our study period was limited to 39 years, whilst the study advancing this hypothesis [18] cited data stemming from more than one century apart. Therefore, the present results do not contradict the hypothesis of a progressively earlier-occurring peak in the past, but they indicate that any such development does not seem to be ongoing, at least in the Austrian population. Despite lacking any time trend, the location of the peak showed considerable (albeit unsystematic) variation over the study period. This might, on the one hand, be a result of using only moderate smoothing. On the other hand, it might offer an explanation for varying results of prior research concerning the exact location of the highest suicide incidence within the annual cycle.

For the reasons elaborated above, we consider our data set as quite typical. This applies also to our results, with one exception: Strength of suicide seasonality was found to be stable when the association with absolute suicide numbers was corrected for. This association was found in all subsamples, except for hanging in women where it could not be assessed due to lack of a trend in absolute suicide numbers. Only two subgroups showed marginal decreases in amplitude, namely men who died by drowning, and women who used the method of hanging. Drowning is a very uncommon method and therefore not a main contributor to overall suicide seasonality. Hanging in women did not show strong seasonality; hence, annual seasonality is mainly driven by men who take their lives by hanging. Since no time trends were found for this latter method, seasonality seems to be a stable phenomenon, at least in the Austrian population.

Furthermore, the amplitude of seasonal patterns showed considerable variation over the study period. Previous research did not assess the strength of seasonality continuously. Instead, time series were split into intervals, and the interval-specific results also showed considerable variation (as can be seen from the range of numbers mentioned above). We therefore assume that both variation in the strength of seasonality and an association of strength with absolute suicide numbers might also be found in other populations.

### Study Limitations

The present findings are based on population statistics of a single country. This is true for most studies in suicide seasonality research; the only comprehensive cross-national study was performed in 1995 [33]. Although we found sample characteristics to be comparable to those of other countries, it still remains to be shown that the current conclusions hold for other countries.

### Implications

It has been argued in previous studies that decreases in suicide seasonality might indicate that some interventions are able to reduce the risk of suicide at times of high risk [34] or that such decreases might be related to a smaller proportion of depressive suicides [10]. This was not confirmed by our results, as there was evidence that the seemingly decreasing strength of seasonality might be a result of its association with absolute suicide numbers. Therefore, a possibly lowered rate of depressive suicides would lower absolute suicide numbers, but not the strength of suicide seasonality.

In summary, our study demonstrates the usefulness of complex demodulation to assess the strength of suicide seasonality. This method allows a continuous estimation of amplitude during a given study period, which facilitates incorporating the association of strength of seasonality with absolute suicide numbers. We could show this association to be significant, and, when considering this fact, we demonstrated that seasonality of suicides seems to be stable in Austria. Although replications in other countries are needed to assess the generalizability of these findings, incorporating the methodological approach of this study seems to have potential to yield a clearer understanding of changes in seasonal patterns of suicide incidence.
